# Synthesis and Properties of SrTiO_3_ Ceramic Doped with Sm_2_O_3_

**DOI:** 10.3390/ma14247549

**Published:** 2021-12-09

**Authors:** Maxim V. Zdorovets, Bekzat A. Prmantayeva, Artem L. Kozlovskiy

**Affiliations:** 1 Laboratory of Solid State Physics, The Institute of Nuclear Physics, Ibragimov Str., Almaty 050032, Kazakhstan; mzdorovets@gmail.com; 2 Engineering Profile Laboratory, L.N. Gumilyov Eurasian National University, Satpayev Str., Nur-Sultan 010008, Kazakhstan; jan_erke_2002@mail.ru; 3 Department of Intelligent Information Technologies, Ural Federal University, Mira Str. 19, 62000 Ekaterinburg, Russia; 4 Institute of Geology and Oil and Gas Business, Satbayev University, Almaty 050032, Kazakhstan

**Keywords:** ferroelectrics, doping, nanostructures, purification of aqueous media, dielectric constant, samarium oxide

## Abstract

The aim of this work was to study the effect of samarium oxide doping on a SrTiO_3_ perovskite ceramic. After analyzing the data obtained on the morphological features of the synthesized structures, it was found that an increase in the dopant concentration led not only to a change in the morphological features, but also in the density of the ferroelectrics. Using the X-ray diffraction method, it was found that doping with Sm_2_O_3_ led to the formation of a multiphase system of two cubic phases of SrTiO_3_ and Sm_2_O_3_. At the same time, an increase in the concentration of Sm_2_O_3_ dopant led to a change in the crystallinity degree, as well as deformation of the structure. Evaluation of the efficiency of use of synthesized ferroelectrics as catalysts for purification of aqueous media from manganese showed that an increase in the concentration of Sm_2_O_3_ dopant led to an increase in purification efficiency by 50–70%.

## 1. Introduction

One of the fundamental problems of ferroelectrics based on titanates is the determination of the laws of influence of various dopants on physicochemical, structural, and optical properties [1,2,3]. At the same time, the choice of dopants in most cases is due to the desire to change properties in order to increase resistance to external influences, increase productivity efficiency, and expand the areas of practical application. As is known, alkaline earth titanates of the SrTiO_3_, BaTiO_3_, and CaTiO_3_ types have a number of unique properties that have opened them to widespread use as capacitor materials, microelectronic devices, catalysts, etc. [4,5,6]. In turn, doping them with various oxides, as well as the possibility of obtaining nanostructured ferroelectrics, makes it possible to make significant changes in their properties, which opens up new prospects for the practical use of ferroelectrics and the expansion of their applications [7,8]. At the same time, ceramics based on strontium and barium titanates have high dielectric constant indices, and doping in these cases can significantly increase these indices, while reducing the threshold value of phase transition temperature, as well as changing the temperature dependence of phase transitions. In turn, the choice of samarium oxide as a dopant, which has unique dielectric and magnetic properties, as well as high resistance to external effects and corrosion, allows the expansion of the properties of titanates due to the formation of solid solutions of introduction or substitution [9,10,11,12,13,14,15]. 

In recent years, ferroelectrics have increasingly been used as a basis for various catalysts for hydrogen production, decomposition of organic dyes, purification of aqueous media, etc. [15,16,17,18,19,20]. Interest in this field of application is due to the increasing problem of contamination of aquatic media with various poisonous substances, products of petrochemical and metallurgical production, heavy metals, and dyes. Their accumulation in aquatic media leads to poisoning of flora and fauna, and the mutagenic accumulation effect can have a negative effect not only on this generation of living organisms, but also on subsequent ones by causing various mutations, etc. One method of removing harmful substances and heavy metals from aqueous media is the method of their absorption on the surface of catalysts or their decomposition into harmless constituents [21,22,23]. In most cases, grain sizes and catalyst phase composition play an important role in the decomposition rate or purification degree of aqueous media. As is known, grain size reduction results in a significant increase in specific surface area, which plays the most important role in decomposition and absorption [24,25]. The band gap and refractive index, which are responsible for the electronic structure of the catalyst and the efficiency of photoelectron output in the case of photocatalytic reactions, also play an important role. In turn, imparting magnetic properties to ferroelectrics or increasing their resistance to corrosion under the action of aqueous media will significantly increase their service life, as well as the efficiency of extraction from the medium without loss of catalyst weight [26,27,28,29,30].

In summary, the main objective of this work was to investigate the effect of samarium oxide doping of the perovskite structure of a SrTiO_3_ ferroelectric, as well as their use as catalysts for the purification of aqueous media from manganese. The choice of the structure of ceramics based on SrTiO_3_ was due to their unique physicochemical properties, as well as the prospects for application not only in microelectronics and as a basis for catalysts. The choice of Sm_2_O_3_ as a dopant was due to its unique optical properties, which lead to the possibility of creating additional absorption bands in the structure of ceramics, as well as a change in the electron density and an improvement in the crystallinity degree of ceramics. The relevance of this study lies not only in the search for solutions to the fundamental issue of studying the change in the structural properties of titanates as a result of their doping, but also in expanding the range of their practical use.

## 2. Experimental Part

### 2.1. Reagents Used for Synthesis

Titanium oxide–anatases (TiO_2_), strontium carbonate (SrCO_3_), and samarium oxide (Sm_2_O_3_) manufactured by Sigma Aldrich Ltd. (Saint Louis, MO, USA) were selected as the original component. To obtain the original sample without doping, the mixing components TiO_2_ and SrCO_3_ were produced in equal shares. The doping was carried out by adding Sm_2_O_3_ in the fractions of x = 0.10, 0.15, 0.20, 0.25 mol and subtracting this fraction of the mass of the mixture of TiO_2_ and SrCO_3_. The chemical purity of the reagents was 99.95%. 

### 2.2. Method of Obtaining Nanostructured Ceramics

As a method for producing nanostructured ceramics based on strontium titanate doped with samarium oxide, the method of solid-phase mechanochemical synthesis combined with thermal treatment in an air atmosphere at a temperature of 1100 °C was used. The mechanochemical synthesis was carried out by grinding the mixtures obtained in a PULVERISETTE 6 planetary mono mill (Fritsch Laboratory Instruments, Idar-Oberstein, Germany). The grinding conditions were: 400 rpm for 5 h. The samples were ground in a glass with tungsten carbide balls 10 mm in diameter, the use of which made it possible to avoid the appearance of impurity metal or metal oxide inclusions in ceramics. 

Annealing was carried out in an SNOL (Lithuania) muffle furnace at a temperature of 1100 °C for 8 h, at a heating rate of 10 °C/min. The samples were annealed in ceramic crucibles that could withstand heating up to 1500 °C. The cooling of the samples to room temperature was conducted in the furnace for 24 h. 

### 2.3. Study of the Morphological Features of Ceramics

The study of morphological features and grain sizes, as well as their shape, was carried out using visualization on a JEOL–7500F scanning electron microscope (JEOL, Akishima, Japan). The dynamics of changes in the shape and size of grains was determined by analyzing the obtained SEM images of synthesized ceramics depending on the dopant concentration. 

Determination of the grain size was also carried out using the method of laser diffraction implemented using an ANALYSETTE 22 NanoTech (Fritsch NanoTec, Idar-Oberstein, Germany) device. 

### 2.4. Determination of the Phase Composition and Crystallographic Parameters

The effect of doping on the phase composition of the synthesized ceramics was evaluated using the powder X-ray diffraction method implemented on a D8 Advance Eco X-ray diffractometer (Bruker, Mannheim, Germany). A copper tube with a wavelength of Cu-kα = 1.54 Å (Cu-kα1 = 1.54056 Å and Cu-kα2 = 1.544398 Å) was used as an X-ray source; diffraction patterns were taken in the Bragg–Brentano geometry in the angular range of 2θ = 20–100°, with a step of 0.03°; and the time of the spectrum set was 1 s at a point. Before processing and analyzing the obtained diffraction patterns, Cu-kα2 was subtracted from the spectra by a mathematical operation. 

The phase composition was determined by selecting the corresponding phases from the PDF-2 (2016) database; the phase selection was based on the coincidence of the positions of the diffraction maxima of the experimentally obtained diffraction patterns with the results of the database with a coincidence of the positions with a probability of more than 85–90%. The contributions of each phase to the composition of doped ceramics were estimated by recalculating the contributions of each phase using Formula (1) [31]:

(1) $$V_{admixture}=\frac{RI_{phase}}{I_{admixture}+RI_{phase}},$$

where *I_phase_* is the average integrated intensity of the main phase of the diffraction line, *I_admixture_* is the average integral intensity of the additional phase, and *R* is the structural coefficient (equal to 1.45).

The structural deformation degree was determined by calculating the ratio of changes in the values of interplane distances for the main diffraction lines as a result of an increase in the dopant concentration.

The porosity of ceramics was determined by calculating changes in the density of the ceramics and the volume of the crystal lattice as a result of changes in the phase composition and the concentration of the dopant. 

Determination of the crystallinity degree of ceramics was carried out by assessing the area of diffraction lines and their subsequent approximation using pseudo-Voigt functions, as well as the calculation of the contribution of disordering regions and amorphous inclusions in the ceramics.

### 2.5. Study of the Optical and Dielectric Properties of Ceramics

To study the doping effect on the optical properties of ceramics, the UV–vis spectroscopy method was used, which allowed us to evaluate the changes in the transmission and absorption capacity of ceramics, as well as to determine the band gap reflecting the change in electron density. The UV–vis spectra were captured in the wavelength range from 300 to 800 nm, and a Jena Specord-250 BU analytical spectrophotometer was used to capture the spectra. The spectra were recorded using the diffuse reflection method using an integrating sphere. BaSO_4_ was used as a standard. The resolution was 1 nm, and the scan rate was 5 nm/s.

### 2.6. Determination of the Manganese Absorption Efficiency

To determine the effect of the dopant concentration and, consequently, the phase composition of the ceramics, on the purification efficiency of the model medium from manganese, a standard method for determining changes in the optical density of the model solution in the wavelength range of 440–460 nm was used. The concentration of manganese in the model solution was 22 mg/dm^3^. The absorption efficiency was determined at different concentrations of ceramics in the solution, which were 0.001, 0.005, and 0.01 g; the absorption time was 1 h.

The manganese absorption efficiency was determined using Formula (2) [32]:

(2) $$Absorption\_effeciency=\left(\frac{C_{0}-C}{C_{0}}\right)*100\%,$$

where *C*_0_ and *C* are the concentration of manganese in the initial solution and after absorption.

## 3. Results and Discussion

**Figure 1:**
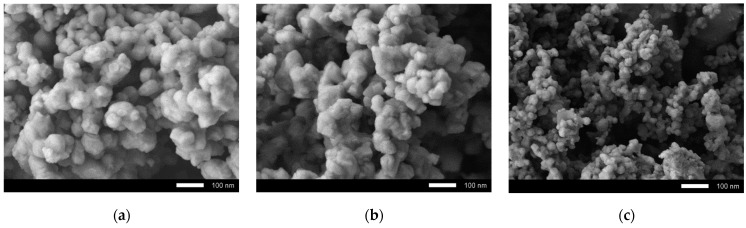

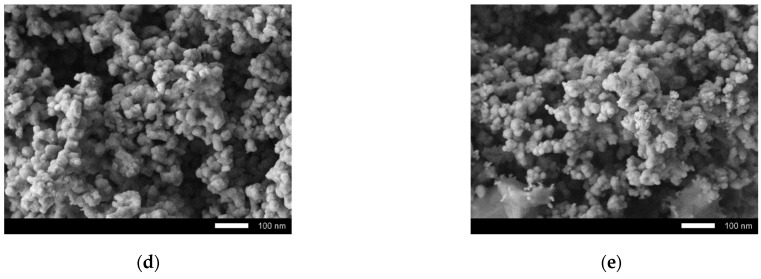
SEM images of synthesized ceramics: (**a**) pristine sample; (**b**) x = 0.10; (**c**) x = 0.15; (**d**) x = 0.20; (**e**) x = 0.25.

Figure 1 shows the results of the study of the morphological features of synthesized ceramics depending on the dopant concentration. As can be seen from the presented SEM images, in the case of the initial undoped ceramics, the morphology was represented by conglomerates of grains, spherical or spherelike in shape, the size of which were 70–90 nm. It can be seen that the formed grain conglomerates had a dendritelike structure consisting of 10–20 grains soldered together. For samples with a concentration of Sm_2_O_3_ equal to x = 0.10 mol, there were no significant differences from the original samples. However, significant changes in the morphological features of the grains were manifested with an increase in the concentration of Sm_2_O_3_, and were expressed in the form of a decrease in the grain size to 30–50 nm, as well as the formation of dendrites with a more developed branch structure. A decrease in the grain size leads to an increase in the specific surface area, as well as the formation of a large number of grain boundaries, which can play an important role in absorption reactions. As is known, the smaller the grain size, the larger the specific surface area of its surface, thereby a larger area is involved in absorption or decomposition reactions. Increasing the specific surface area is the basis of one of the ways to increase the efficiency of catalytic reactions. 

**Figure 2:**
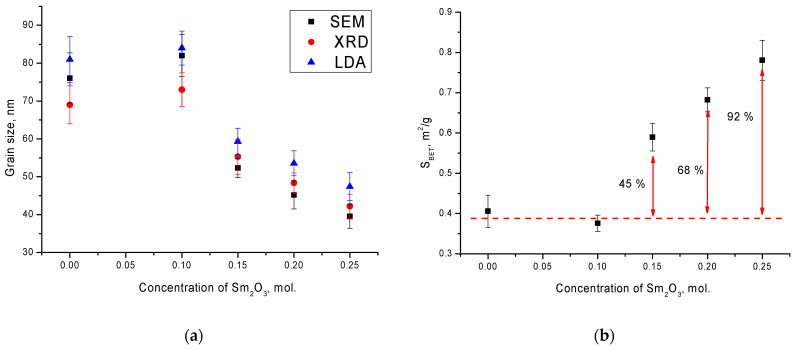
(**a**) Results of a comparative analysis of grain sizes; (**b**) results of the specific surface area determination.

Figure 2a shows the results of a comparative analysis of grain sizes determined using scanning electron microscopy (SEM), X-ray diffraction (XRD), by sizing using the Scherer equation, and laser diffraction analysis (LDA). As can be seen from the presented data, in the case of the initial ceramics and in those doped with a Sm_2_O_3_ concentration equal to x = 0.10, there was a good agreement of the grain sizes determined by three different methods, with an accuracy within the error limits. In this case, as shown in the SEM images, at a Sm_2_O_3_ concentration above x = 0.10, a decrease in the grain size was observed. In this case, there was a slight deviation in the results of grain sizes determined by the LDA method, which was associated with the formation of compressed agglomerates of irregular shape, which led to a slight increase in the average value of grain sizes. 

Figure 2b shows the results of the specific surface area (*S*_BET_) determination, which was calculated using the Brunauer–Emmett–Teller method and Formula (3):

*S*_BET_ = 6/(*ρ*_X_ · *D*_BET_), (3)

where *ρ*_X_ is the density of ceramics, and *D*_BET_ is the grain size.

As can be seen from the presented data, a decrease in the grain size led to a significant increase in the *S*_BET_ value for ceramics with a Sm_2_O_3_ dopant concentration above x = 0.10.

**Figure 3:**
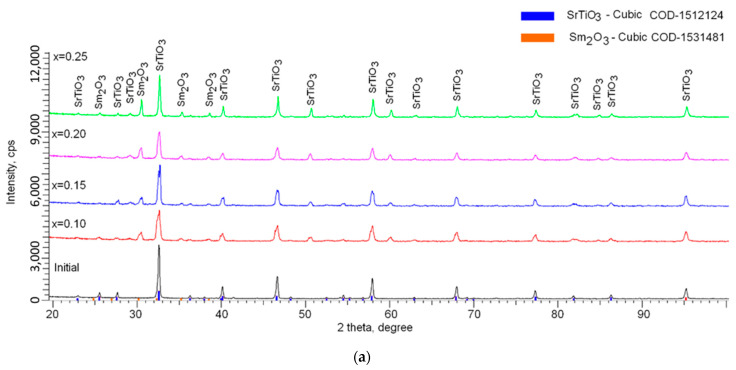

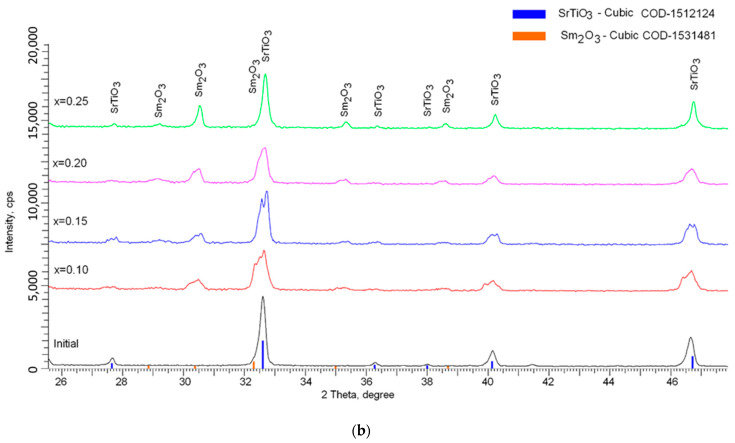
(**a**) Data of X-ray diffraction patterns of synthesized ceramics depending on the concentration of the Sm_2_O_3_ dopant; (**b**) detailed representation of changes in the position and shape of the main reflexes depending on the dopant concentration.

Figure 3 shows the results of the phase analysis performed on the basis of the obtained X-ray diffraction patterns. In the initial state, the synthesized ceramics consisted of a cubic phase of SrTiO_3_ of a perovskite-like structure, without the presence of impurities in the form of oxides or carbides of titanium or strontium. The absence of impurities was due to the fact that under the selected conditions of heat treatment after mechanochemical synthesis, the structure of perovskite of the ABO_3_ type was formed by phase transformations of titanium oxide and strontium carbonate, with complete carbon burnout [33,34]. At the same time, the analysis of the shape and position of the diffraction lines indicated the presence of deformation distortions and stresses in the structure caused by the synthesis process and subsequent sintering [33,34,35]. According to the estimation of the crystal lattice parameters performed using the Nelson–Taylor technique implemented in the DiffracEVA v.4.2 program code, it was found that for the initial sample, the crystal lattice parameter was *a* = 3.8797 Å, the lattice volume was V = 58.40 Å^3^, and the deviation of parameter *a* from the reference value was 0.65%.

For samples doped with Sm_2_O_3_, one of the characteristic changes was the appearance of a number of new diffraction reflexes in the region of 2θ = 25–40°, the intensity of which and, consequently, the contribution, increased with an increase in dopant concentration. According to the phase analysis, these diffraction reflexes corresponded to the cubic phase of Sm_2_O_3_ with the spatial syngony Ia-2(206). At the same time, the evaluation of diffraction reflexes, their position, and shape showed the absence of any phases other than SrTiO_3_ and Sm_2_O_3_, which indicated that doping led to the formation of a multiphase superposition system of two phases. In the case of samples with a dopant concentration of x = 0.10 and 0.15, a stratification of the main diffraction reflexes characteristic of mechanical eutectic was observed. This behavior of diffraction reflexes indicated that an increase in the dopant concentration led to an increase in the superposition of two phases insoluble in each other. An increase in the concentration above x = 0.15 led to the absence of stratification of diffraction reflexes, the formation of a stable structure with a high crystallinity degree, and small deformations of the structure [36,37,38].

The presence of double peaks at low concentrations of the Sm_2_O_3_ dopant was due to the similarity of the structural characteristics (see Figure 3b), which made it possible to identify these phases. At the same time, at low concentrations, the dopant had a significant effect on the structure deformation due to the sintering and implantation processes. This effect led to the asymmetry of diffraction reflections, as well as their stratification. At high concentrations of Sm_2_O_3_, the process of substitution and insertion was carried out to the end, which led to an increase in the structure ordering, which was evidenced by an increase in the crystallinity degree. An increase in the Sm_2_O_3_ dopant concentration above x = 0.15 led to an increase in the intensity of diffraction reflections characteristic of Sm_2_O_3_, which in turn indicated an increase in the contribution of the Sm_2_O_3_ phase to the structure of ceramics. The absence of peaks characteristic of complex phases also indicated that doping led to the formation of a solid solution of a mixture of two phases, insoluble in each other, without the formation of intermediate compounds. 

**Figure 4:**
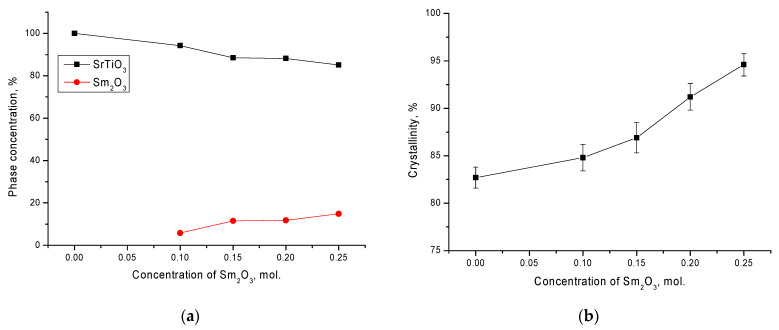
(**a**) Results of the phase composition of ceramics depending on the dopant concentration; (**b**) results of changes in the crystallinity degree.

Figure 4a shows the results of determining the phase composition using Formula (1). As can be seen from the data presented, a change in the dopant concentration led to the formation of the Sm_2_O_3_ phase in the structure of ceramics from 5 to 15%, while an increase in the concentration from 0.15 to 0.20 led to a slight increase in the Sm_2_O_3_ phase in the structure. This behavior of the phase composition may have been due to the eutectic compositions of ceramics. An increase in the Sm_2_O_3_ phase, as shown by the data of changes in the crystallinity degree, led to an increase in structural orderings and a decrease in the concentration of amorphous inclusions and disordered regions. In addition, at dopant concentrations above 0.15, the crystallinity degree increased significantly for ceramics with no delamination effect on their diffraction patterns (see Figure 4b). The crystallinity degree was estimated by comparing the contributions of the areas of the main reflections to the area of background radiation associated with the presence of regions of disorder in the structure.

Table 1 shows the data of changes in the crystallographic parameters of the studied ceramics depending on the dopant concentration. The density of the material was calculated using Formula (4):

(4) $$p=\frac{1.6602\sum AZ}{V_{0}},$$

where *V*_0_ is the unit cell volume, *Z* is the number of atoms in a crystal cell, and *A* is the atomic weight of atoms. The integral porosity of the samples under study was found according to Formula (5):

(5) Pdil=(1−pp0)∗100%

where *p*_0_ is the density of the reference sample.

As can be seen from the data presented, an increase in the dopant concentration, and therefore the contribution of the Sm_2_O_3_ phase, led to a change in the crystal lattice parameters, as well as a decrease in the porosity of the ceramics. At the same time, a decrease in porosity was associated with an increase in the density of ceramics associated with an increase in structural ordering and a decrease in the concentration of disordered regions in the structure of ceramics. It should also be noted that the most pronounced changes in the density of ceramics were observed for structures with a dopant concentration above x = 0.15.

**Figure 5:**
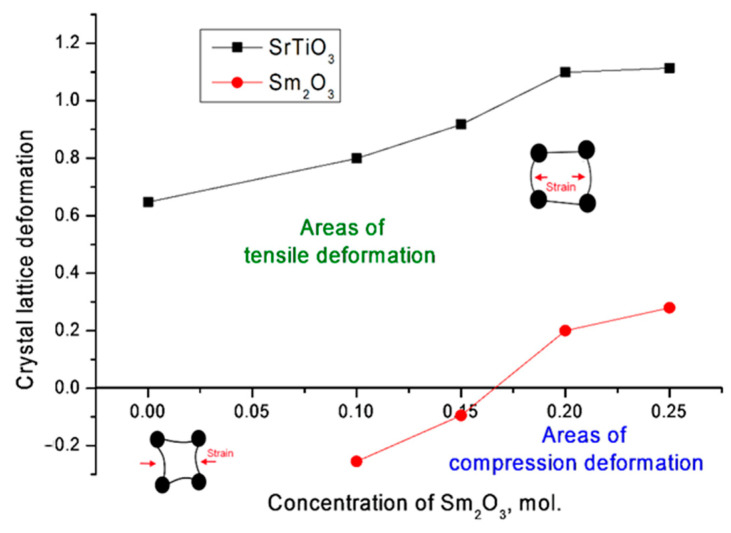
Results of changes in crystal lattice deformation. The insets show schematic images of the crystal lattice deformation caused by a change in the dopant concentration.

At the same time, according to the estimation of the deformation of the crystal lattice, the main contribution to its distortion for the SrTiO_3_ phase was made by tensile stresses, while for the Sm_2_O_3_ phase at low concentrations, the deformation occurred due to the compression of the lattice, which was due to the presence of two phases in the structure of the ceramics and mechanical eutectic. An increase in the concentration of Sm_2_O_3_ and, accordingly, the contribution of the cubic phase of Sm_2_O_3_ also led to a transition to deformation processes of stretching the crystal lattice (see Figure 5). This behavior of deformation contributions may have been due to the absence of the possibility of formation of complex phases under the selected synthesis conditions, as well as partial substitutions of strontium or titanium atoms with samarium atoms, which can occupy both positions in the lattice nodes and in the interstices.

**Figure 6:**
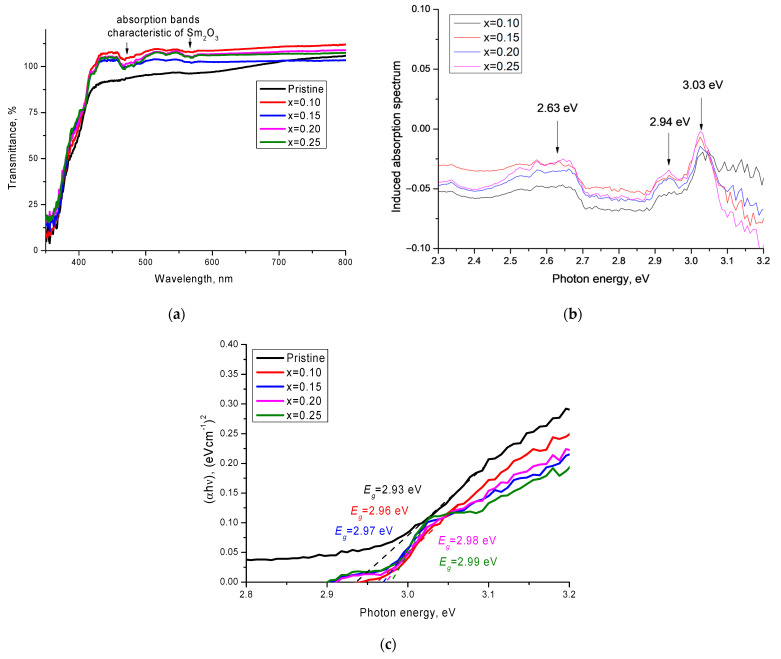
(**a**) Transmission spectrum data for the studied ceramics; (**b**) induced absorption spectra resulting from doping; (**c**) Tauc plots for determination of the band gap.

Figure 6a shows the results of changes in the optical properties of the ceramics depending on the dopant concentration, presented in the form of optical transmission spectra. The main changes in the optical properties of the synthesized ceramics can be expressed in the form of a shift of the fundamental absorption edge, which characterized the change in the electron density and the band gap, as well as the appearance of several additional absorption bands in the 450–600 nm region. The logarithm of the ratio of the optical transmission spectra (T_0_(λ)/T(λ)) and their representation in the energy scale allowed us to obtain a more pronounced picture of the induced absorption spectra, as well as to determine their maxima (see Figure 6b). According to the data obtained, an increase in the concentration of the Sm_2_O_3_ dopant led to the formation of and an increase in the intensity of induced absorption bands, with maxima at 2.63 eV, 2.94 eV, and 3.03 eV. The presence of such maxima was associated with the contribution of samarium oxide and the formation of additional absorbing centers.

The determination of the band gap (*E_g_*) was carried out by analyzing the obtained UV–vis spectra and Tauc plots using Formula (6):

(6) $$\alpha =A{\left(h\nu -E_{g}\right)}^{1/2},$$

where *A* is a constant, and *hν* is the photon energy.

The results of change in the band gap shown in Figure 6c showed that an increase in the concentration of Sm_2_O_3_ led to a shift in the fundamental absorption edge and an increase in the band gap from 2.93 eV to 2.99 eV at the maximum value of the dopant concentration. This behavior of the change in the band gap was associated with the electronic structure of samarium oxide, the formation of the phase of which led to a change not only in the structural properties, but also in the optical ones.

**Figure 7:**
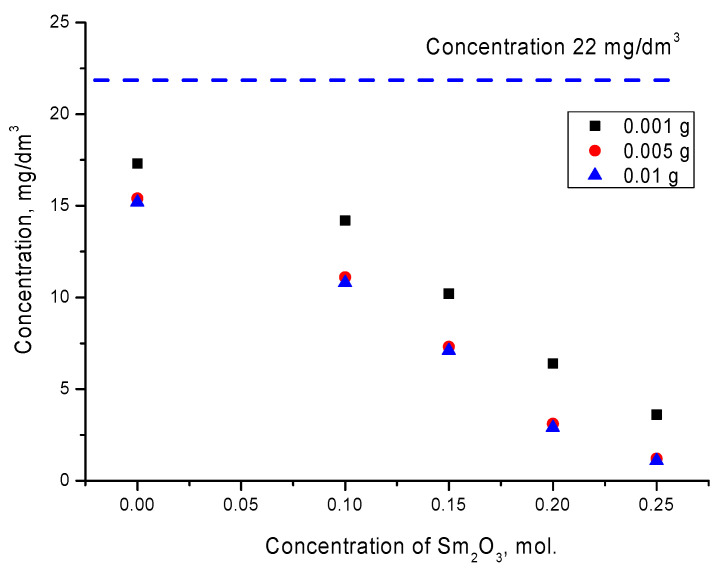
Results of changes in the concentration of manganese as a result of the absorption reaction for the studied ceramics. The dotted blue line indicates the concentration of manganese without dye.

Figure 7 shows the results of the change in the concentration of manganese in the model solution after the absorption reaction for 1 h for different concentrations of ceramics placed in the solution. The concentration was calculated based on the change in the optical density of the solution. As can be seen from the data presented, the concentration of manganese in the solution for undoped ceramics was quite high, and exceeded 15–17 mg/dm^3^. At the same time, an increase in the content of ceramics from 0.001 g to 0.005 g led to a slight decrease in the concentration, which indicated the absorption efficiency due to an increase in the mass of the absorbent. However, an increase in the mass of ceramics to 0.01 g did not lead to significant changes in the manganese concentration decrease in the solution, which indicated the saturation effect. 

**Figure 8:**
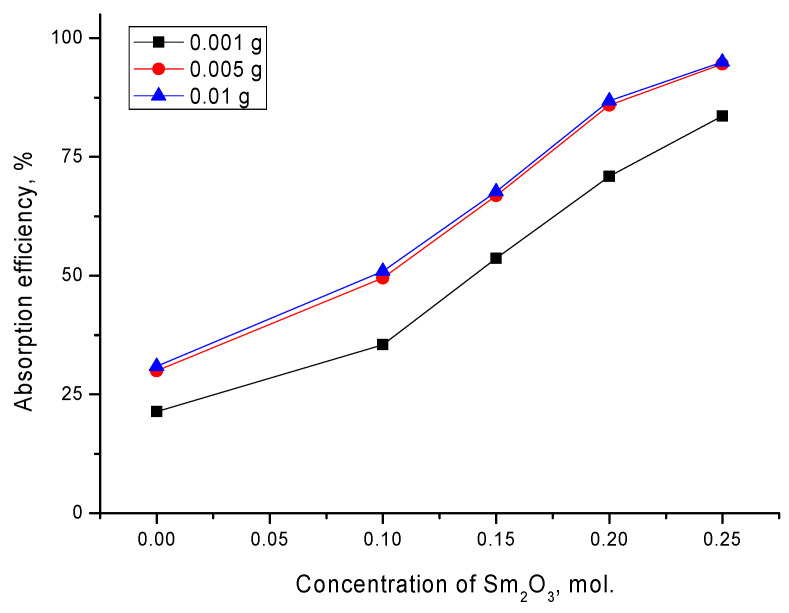
Results of the manganese absorption efficiency depending on the dopant concentration.

For doped ceramics, according to the obtained optical density results, it was clear that an increase in the dopant content, which led to a change not only in grain sizes, but also in structural and optical properties [28,30], led to a decrease in the concentration of manganese and an increase in the absorption efficiency. At the same time, as in the case of the initial ceramics, an increase in the content from 0.005 g to 0.01 g did not lead to a significant increase in the absorption efficiency. Figure 8 shows the results of the absorption efficiency for the studied ceramics, calculated using Formula (2).

According to the data obtained, it can be seen that in the case of initial ceramics, the purification efficiency was no more than 23–27%, depending on the content of ceramics in the solution. Doping of the ceramics with samarium oxide with a concentration of x = 0.1 led to an increase in the efficiency of 15–20% compared to the initial ceramics, while an increase in the content of ceramics in the solution from 0.001 g to 0.005 g and 0.01 g also led to an increase in the absorption efficiency by 15%. It is worth noting that such an increase in efficiency with an increase in the content from 0.001 g to 0.005 g and 0.01 g was preserved for all doped ceramics. The most effective were ceramics with a dopant concentration x = 0.2–0.25 mol, for which the absorption efficiency was more than 70–80% for a content of 0.001 g, and more than 90% for a concentration of 0.005 g and 0.01 g. In this case, we concluded that the use of these types of ceramics allowed the absorbance of almost all of the manganese from the aqueous medium, reducing its concentration to 3–5% of the initial value. At the same time, tests for long-term operation, consisting of the reuse of these ceramics for purification from manganese, showed that doped ceramics retained their efficiency for 5–7 consecutive cycles, with a slight decrease in efficiency after 5 cycles by 5–10%, while undoped ceramics lost their absorption efficiency after 3 cycles.

## 4. Conclusions

The paper presented the results of a study of the effect of doping with samarium oxide of ceramics based on strontium titanate obtained by solid-phase synthesis followed by thermal annealing at a temperature of 1100 °C. During the conducted studies, it was found that an increase in the dopant concentration led to the formation of a superposition system of two phases of SrTiO_3_ and Sm_2_O_3_, with the dominance of the cubic SrTiO_3_ phase in the structure. It was found that an increase in the contribution of the Sm_2_O_3_ phase led to an increase in structural orderings and a decrease in the concentration of amorphous inclusions and disordered regions. During the study of the optical properties of ceramics based on transmission spectra, it was found that an increase in the concentration of the Sm_2_O_3_ dopant led to the formation and increase in the intensity of induced absorption bands with maxima at 2.63 eV, 2.94 eV, and 3.03 eV. The evaluation of the efficiency of the use of synthesized ferroelectrics as catalysts for the purification of aqueous media from manganese showed that an increase in the concentration of the Sm_2_O_3_ dopant led to an increase in the purification efficiency by 50–70%. 

## Figures and Tables

**Table 1:** The results of the crystallographic parameters of the studied ceramics.

Parameter	Phase	Concentration of Sm_2_O_3_, mol				
		0	0.10	0.15	0.20	0.25
Lattice parameter, Å	SrTiO_3_	a = 3.8797	a = 3.8738	a = 3.8692	a = 3.8631	a = 3.8615
	Sm_2_O_3_	-	a = 11.0231	a = 11.0054	a = 10.9731	a = 10.9643
Density, g/cm^3^		5.216	5.240	5.259	5.284	5.298
Porosity, %		3.31	3.26	2.72	2.37	1.93

## Data Availability

Not applicable.

## References

[1] Chang C.W., Hu C. (2020). Graphene oxide-derived carbon-doped SrTiO_3_ for highly efficient photocatalytic degradation of organic pollutants under visible light irradiation. Chem. Eng. J..

[2] Bantawal H.U., Shenoy S., Bhat D.K. (2020). Vanadium-Doped SrTiO_3_ Nanocubes: Insight into role of vanadium in improving the photocatalytic activity. Appl. Surf. Sci..

[3] Rena L., Yia X., Tonga L., Zhoub W., Wangac D., Liuac L., Yeacd J. (2020). Nitrogen-doped ultrathin graphene encapsulated Cu nanoparticles decorated on SrTiO_3_ as an efficient water oxidation photocatalyst with activity comparable to BiVO_4_ under visible-light irradiation. Appl. Catal. B Environ..

[4] Wang B., Pu Y., Shi Y., Guo X., Zhang L., Chang L., Li J., Li R., Ji J., Wei T. (2020). Ultralow dielectric loss in Y-doped SrTiO_3_ colossal permittivity ceramics via designing defect chemistry. J. Am. Ceram. Soc..

[5] Singh S.P., Kanas N., Desissa T.D., Johnsson M., Einarsrud M.-A., Norby T., Wiik K. (2019). Thermoelectric properties of A-site deficient La-doped SrTiO_3_ at 100–900 °C under reducing conditions. J. Eur. Ceram. Soc..

[6] Pan W., Cao M., Hao H., Zhonghua Y., Yu Z., Liu H. (2020). Defect engineering toward the structures and dielectric behaviors of (Nb, Zn) co-doped SrTiO_3_ ceramics. J. Eur. Ceram. Soc..

[7] Sandhyarani A., Kokila M., Darshan G., Sharma S., Kavyashree D., Premkumar H., Nagabhushana H. (2020). Photometric features and intense blue light emanation of Nd^3+^ doped SrTiO_3_ based nanophosphor for multi-functional applications. J. Sci. Adv. Mater. Devices.

[8] Wu L., Ji Y., Ouyang B., Li Z., Yang Y. (2021). Self-Powered Light-Temperature Dual-Parameter Sensor Using Nb-Doped SrTiO_3_ Materials Via Thermo-Phototronic Effect. Adv. Funct. Mater..

[9] Gray N.W., Tiwari A. (2011). Dynamic superparamagnetism in cobalt doped Sm_2_O_3_ thin films. J. Appl. Phys..

[10] Ren X., Ma B., Zhang G., Fu G., Yu J., Liu G. (2020). Preparation and properties of MgAl_2_O_4_ spinel ceramics by double-doped Sm_2_O_3_–(Y_2_O_3_, Nb_2_O_5_ and La_2_O_3_). Mater. Chem. Phys..

[11] Yuan L., Ma B., Zhu Q., Wang Z., Li G., Yu J. (2017). Preparation and properties of MgAl_2_O_4_ based ceramics reinforced with rod-like microcrystallines by co-doping Sm_2_O_3_ and La_2_O_3_. Ceram. Int..

[12] Yaru N., Chunhua L., Yan Z., Qitu Z., Zhongzi X. (2007). Study on Optical Properties and Structure of Sm_2_O_3_ Doped Boron-Aluminosilicate Glass. J. Rare Earths.

[13] Wright J., Virkar A.V. (2011). Conductivity of porous Sm_2_O_3_-doped CeO_2_ as a function of temperature and oxygen partial pressure. J. Power Sources.

[14] Ahmadnia-Feyzabad S., Mortazavi Y., Khodadadi A.A., Hemmati S. (2013). Sm_2_O_3_ doped-SnO_2_ nanoparticles, very selective and sensitive to volatile organic compounds. Sens. Actuators B Chem..

[15] Guan L., Li J., Song X., Bao J., Jiang T. (2018). Graphite assisted flash sintering of Sm_2_O_3_ doped CeO_2_ ceramics at the onset temperature of 25 °C. Scr. Mater..

[16] Ismail M., Sazelee N., Ali N., Suwarno S. (2020). Catalytic effect of SrTiO_3_ on the dehydrogenation properties of LiAlH4. J. Alloy. Compd..

[17] Rajkoomar N., Murugesan A., Prabu S., Gengan R.M. (2020). Synthesis of methyl piperazinyl-quinolinyl α-aminophosphonates derivatives under microwave irradiation with Pd–SrTiO_3_ catalyst and their antibacterial and antioxidant activities. Phosphorus Sulfur Silicon Relat. Elem..

[18] Ling J., Wang K., Wang Z., Huang H., Zhang G. (2019). Enhanced piezoelectric-induced catalysis of SrTiO_3_ nanocrystal with well-defined facets under ultrasonic vibration. Ultrason. Sonochem..

[19] Kopač D., Likozar B., Huš M. (2020). How size matters: Electronic, cooperative, and geometric effect in perovskite-supported copper catalysts for CO_2_ reduction. ACS Catal..

[20] He W., Wu X., Li Y., Xiong J., Tang Z., Wei Y., Zhao Z., Zhang X., Liua J. (2020). Z-scheme heterojunction of SnS_2_-decorated 3DOM-SrTiO_3_ for selectively photocatalytic CO_2_ reduction into CH_4_. Chin. Chem. Lett..

[21] Yu X., Lin Y., Liu H., Yang C., Peng Y., Du C., Wu S., Li X., Zhong Y. (2019). Photocatalytic performances of heterojunction catalysts of silver phosphate modified by PANI and Cr-doped SrTiO_3_ for organic pollutant removal from high salinity wastewater. J. Colloid Interface Sci..

[22] Zhang L., Yin J., Wei K., Li B., Jiao T., Chen Y., Zhou J., Peng Q. (2020). Fabrication of hierarchical SrTiO_3_@ MoS_2_ heterostructure nanofibers as efficient and low-cost electrocatalysts for hydrogen-evolution reactions. Nanotechnology.

[23] Coletta V.C., Gonçalves R.V., Bernardi M.I.B., Hanaor D.A.H., Assadi M.H.N., Marcos F.C.F., Nogueira F.G.E., Assaf E.M., Mastelaro V.R. (2020). Cu-Modified SrTiO_3_ Perovskites Toward Enhanced Water–Gas Shift Catalysis: A Combined Experimental and Computational Study. ACS Appl. Energy Mater..

[24] Li Y., Li H., Le S., Bai X., Wang X. (2019). Catalytic conversing heavy concentrations of nitroarenes to aminoarenes over Cu_2_O/Cu/SrTiO_3_ three-phase hybrid under flow conditions. J. Clean. Prod..

[25] Trang T.N.Q., Thu V.T.H. (2021). Effects of the hybrid plasmonic Ag/SrTiO_3_ nanocubes for efficient photo-catalytic of H_2_ generation and RhB decomposition. Sci. Technol. Dev. J..

[26] Wang S., Teramura K., Hisatomi T., Domen K., Asakura H., Hosokawa S., Tanaka T. (2020). Effective Driving of Ag-Loaded and Al-Doped SrTiO_3_ under Irradiation at λ > 300 nm for the Photocatalytic Conversion of CO_2_ by H_2_O. ACS Appl. Energy Mater..

[27] Lei S., Wang A., Xue J., Wang H. (2021). Catalytic ceramic oxygen ionic conducting membrane reactors for ethylene production. React. Chem. Eng..

[28] Wang J.B., Shih W., Huang T. (2000). Study of Sm_2_O_3_-doped CeO_2_/Al_2_O_3_-supported copper catalyst for CO oxidation. Appl. Catal. A Gen..

[29] Shen X., Sasaki K. (2016). Highly redox-resistant solid oxide fuel cell anode materials based on La-doped SrTiO_3_ by catalyst impregnation strategy. J. Power Sources.

[30] Abdi M., Mahdikhah V., Sheibani S. (2020). Visible light photocatalytic performance of La-Fe co-doped SrTiO_3_ perovskite powder. Opt. Mater..

[31] Kozlovskiy A.L., Kenzhina I.E., Zdorovets M.V. (2020). FeCo–Fe_2_CoO_4_/Co_3_O_4_ nanocomposites: Phase transformations as a result of thermal annealing and practical application in catalysis. Ceram. Int..

[32] Ociński D., Jacukowicz-Sobala I., Mazur P., Raczyk J., Kociołek-Balawejder E. (2016). Water treatment residuals containing iron and manganese oxides for arsenic removal from water–Characterization of physicochemical properties and adsorption studies. Chem. Eng. J..

[33] Kaliaguine S., Van Neste A., Szabo V., Gallot J.E., Bassir M., Muzychuk R. (2001). Perovskite-type oxides synthesized by reactive grinding: Part, I. Preparation and characterization. Appl. Catal. A Gen..

[34] Grabowska E. (2016). Selected perovskite oxides: Characterization, preparation and photocatalytic properties—A review. Appl. Catal. B Environ..

[35] Kong L., Zhang T., Ma J., Boey F. (2008). Progress in synthesis of ferroelectric ceramic materials via high-energy mechanochemical technique. Prog. Mater. Sci..

[36] Baláž P., Achimovičová M., Baláž M., Billik P., Cherkezova-Zheleva Z., Criado J.M., Delogu F., Dutková E., Gaffet E., Gotor F.J. (2013). Hallmarks of mechanochemistry: From nanoparticles to technology. Chem. Soc. Rev..

[37] Rendón-Angeles J.C., Matamoros-Veloza Z., Yanagisawa K. (2012). Preparation of selected ceramic compounds by controlled crystallization under hydrothermal conditions. Crystallization-Science and Technology.

[38] Dovlitova L.S., Ivanov D.V., Isupova L.A., Malakhov V.V. (2015). Determination of chemical and phase compositions of mixed oxides with the perovskite-like structure based on strontium titanates by differential dissolution method. J. Struct. Chem..

